# Supplementary figures and images for: BglaTNB6, a tailocin produced by a plant-associated nonpathogenic bacterium, prevents rice seed-borne bacterial diseases
Source: PLoS Pathog. 2024 Oct 18;20(10):e1012645. doi: 10.1371/journal.ppat.1012645 (PMC11524443; doi:10.1371/journal.ppat.1012645)

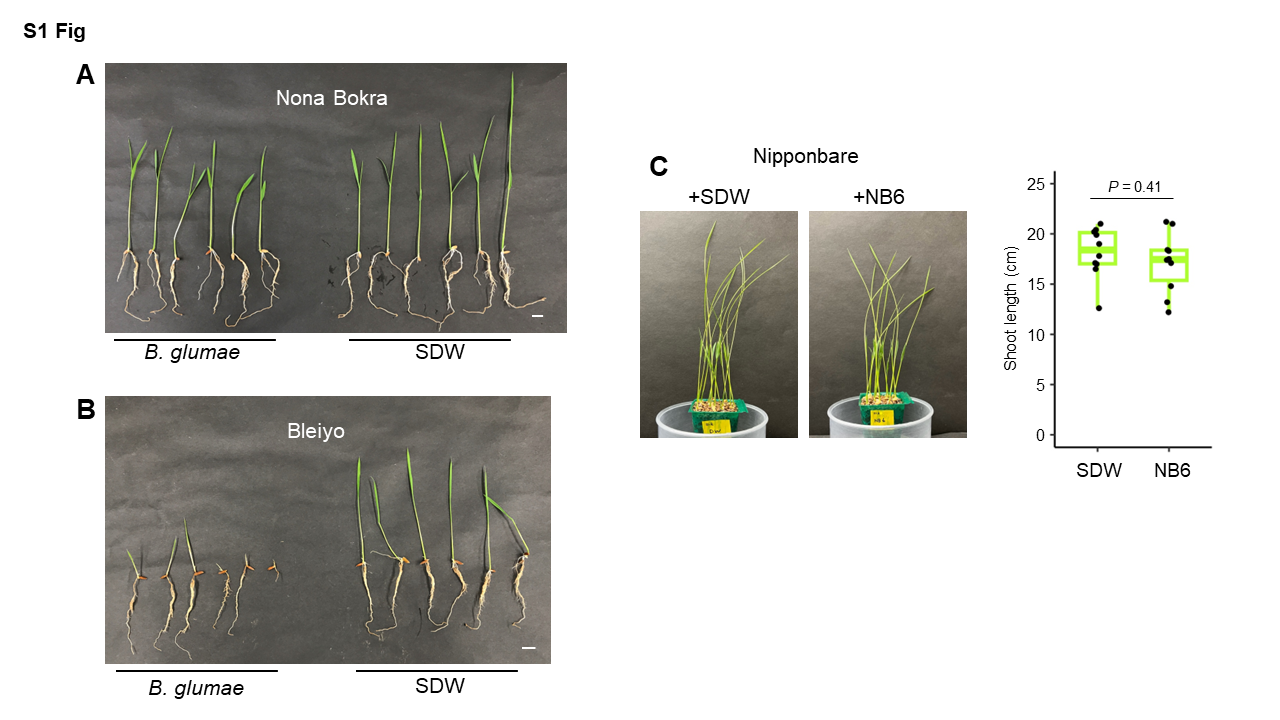

Supplement: S1 Fig — (A) Rice seeds (Nona Bokra) were inoculated with B. glumae or sterilized distilled water (SDW). Photos were taken at 7 days postinoculation (dpi). (B) Rice seeds (Bleiyo) were inoculated with B. glumae or sterilized distilled water (SDW). Photos were taken at 7 dpi. (C) Rice seeds (Nipponbare) were inoculated with B. gladioli NB6 or sterilized distilled water (SDW). Photos were taken at 8 dpi. The box plot shows the shoot length of rice seedlings at 8 dpi: the center lines indicate the medians, the box limits represent the upper and lower quartiles, and the whiskers extend to the maximum and minimum values. Different letters indicate statistically significant differences between groups (Tukey–Kramer test, P < 0.05, n = 10). These experiments were repeated twice, and similar results were obtained. Figures show the results of one representative experiment. (TIF) [file ppat.1012645.s001.TIF]

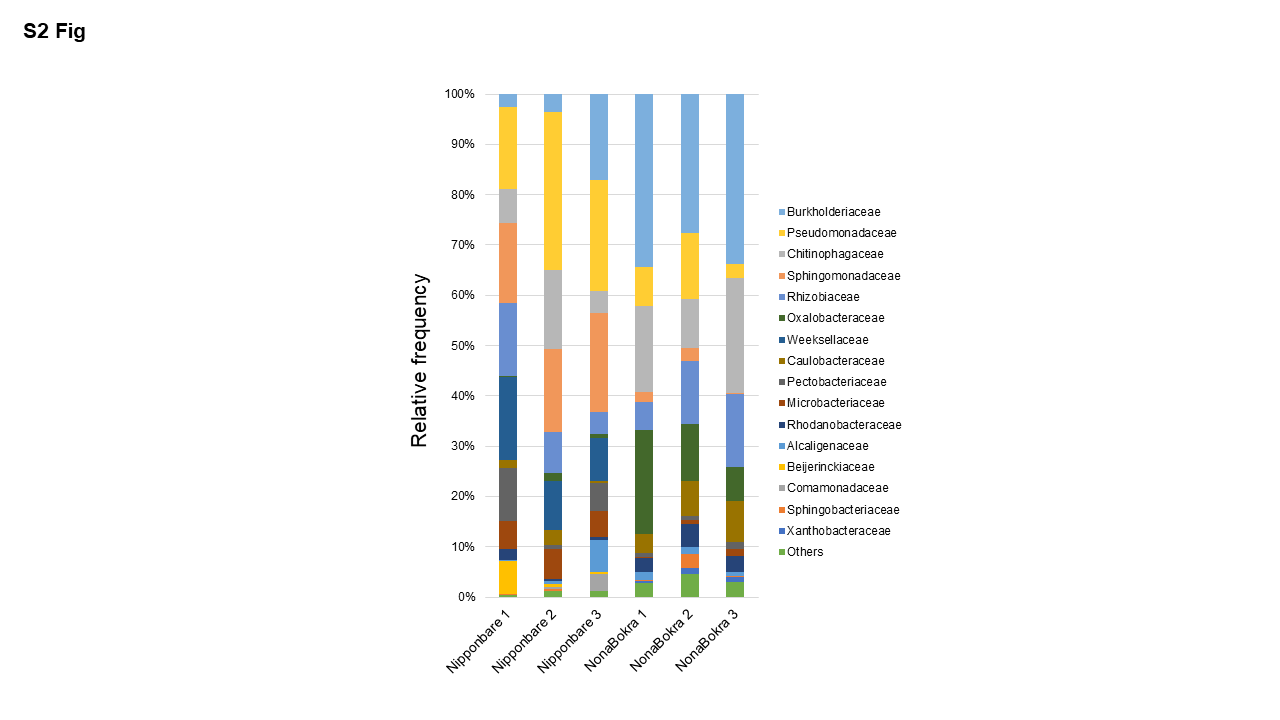

Supplement: S2 Fig — Relative abundance of bacterial families in rice plants of the cultivars Nipponbare and Nona Bokra. (TIF) [file ppat.1012645.s002.TIF]

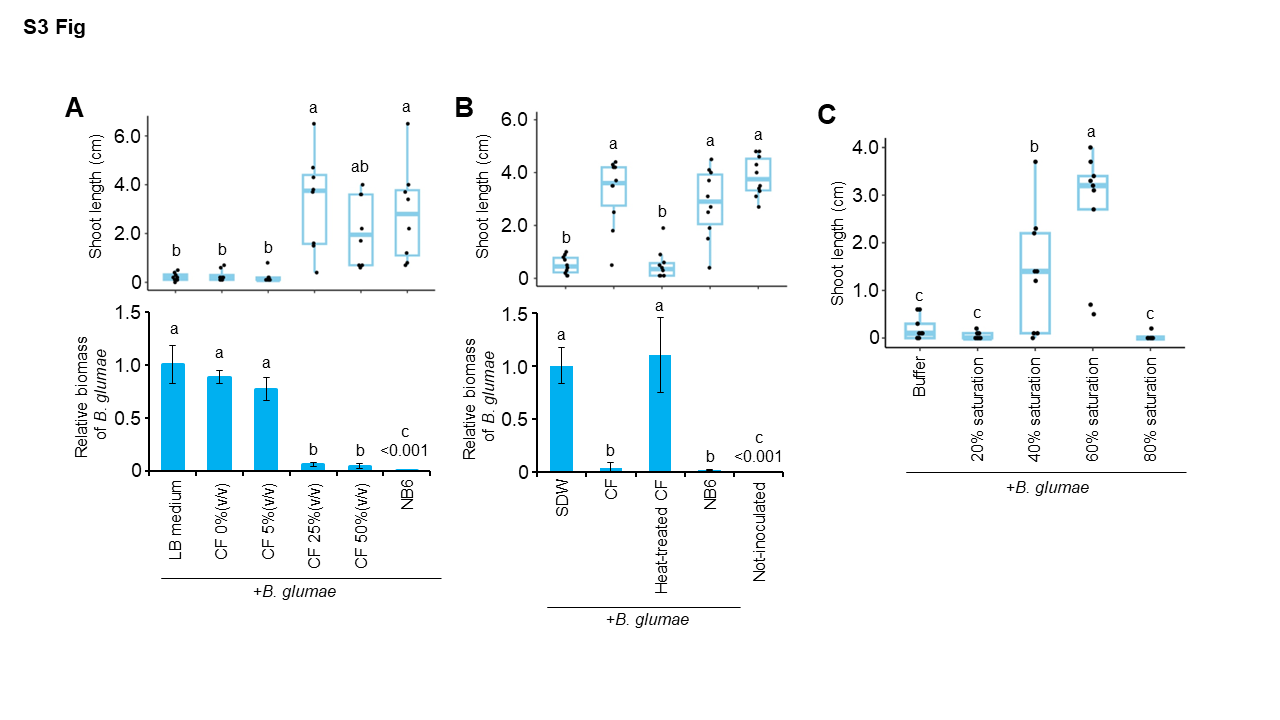

Supplement: S3 Fig — (A) Rice seeds were coinoculated with B. glumae (Bg) and B. gladioli NB6 or its culture filtrate (CF) diluted with liquid LB medium. Upper: the box plot shows the shoot length of rice seedlings at 7 days postinoculation (dpi): the center lines indicate the medians, the box limits represent the upper and lower quartiles, and the whiskers extend to the maximum and minimum values. Different letters indicate statistically significant differences between groups (Tukey–Kramer test, P < 0.05, n = 8). Lower: the bar chart represents the relative biomass of Bg in rice seedlings at 7 dpi, measured by quantitative PCR. Two seedlings were bulked for total DNA extraction. Data are presented as means ± SE of values relative to plants inoculated only with the pathogens. Different letters indicate statistically significant differences between groups (Tukey–Kramer test, P < 0.05, n = 4). (B) Rice seeds were co-inoculated with Bg and NB6, its CF, the CF treated with 95°C for 5 minutes, or sterilized distilled water (SDW). Upper: the box plot shows the shoot length of rice seedlings at 7 dpi, following the same format as in (A). Different letters indicate statistically significant differences between groups (Tukey–Kramer test, P < 0.05, n = 10). Lower: the bar chart represents the relative biomass of Bg in rice seedlings at 7 dpi, following the same format as in (A). Different letters indicate statistically significant differences between groups (Tukey–Kramer test, P < 0.05, n = 4). (C) Rice seeds were co-inoculated with Bg and the fractions of ammonium sulfate precipitation of the CF of NB6 or 50 mM Tris-HCl buffer (pH 7.5). The box plot shows the shoot length of rice seedlings at 7 dpi, following the same format as in (A). Different letters indicate statistically significant differences between groups (Tukey–Kramer test, P < 0.05, n = 9). These experiments were repeated two times, and similar results were obtained. Figures show the results of one representative experiment. (TIF) [file ppat.1012645.s003.TIF]

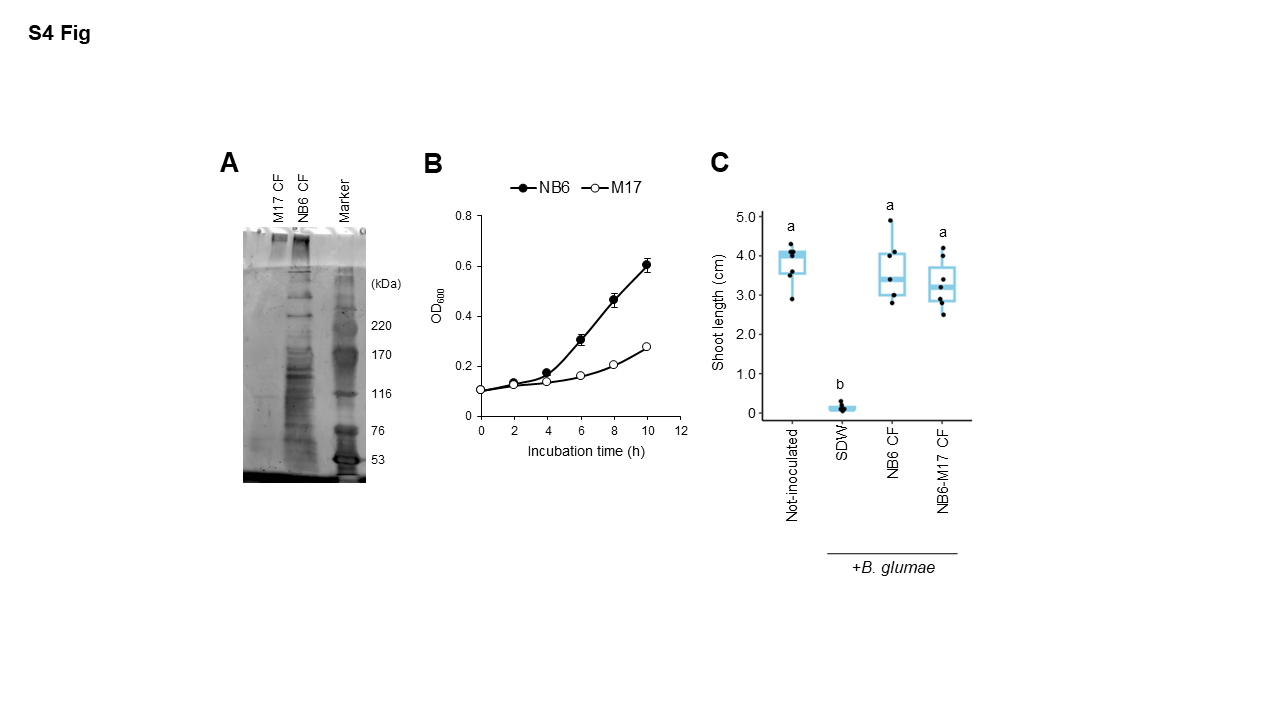

Supplement: S4 Fig — (A) Twenty μl of the 60% saturation fractions obtained from ammonium sulfate precipitation of the culture filtrates (CF) of B. gladioli NB6 and NB6-M17 were resolved by SDS-PAGE. (B) Initial growth of NB6 and NB6-M17 cultured in liquid LB medium. Bacterial growth was measured as OD600 every hour. Error bars represent SE (n = 4). The experiment was repeated twice with similar results obtained. (C) Rice seeds were co-inoculated with B. glumae (Bg) and the CF of NB6, the CF of NB6-M17, or sterilized distilled water (SDW). The box plot shows the shoot length of rice seedlings at 7 days postinoculation: the center lines indicate the medians, the box limits represent the upper and lower quartiles, and the whiskers extend to the maximum and minimum values. Different letters indicate statistically significant differences between groups (Tukey–Kramer test, P < 0.05, n = 7). These experiments were repeated two times, and similar results were obtained. Figures show the results of one representative experiment. (TIF) [file ppat.1012645.s004.TIF]

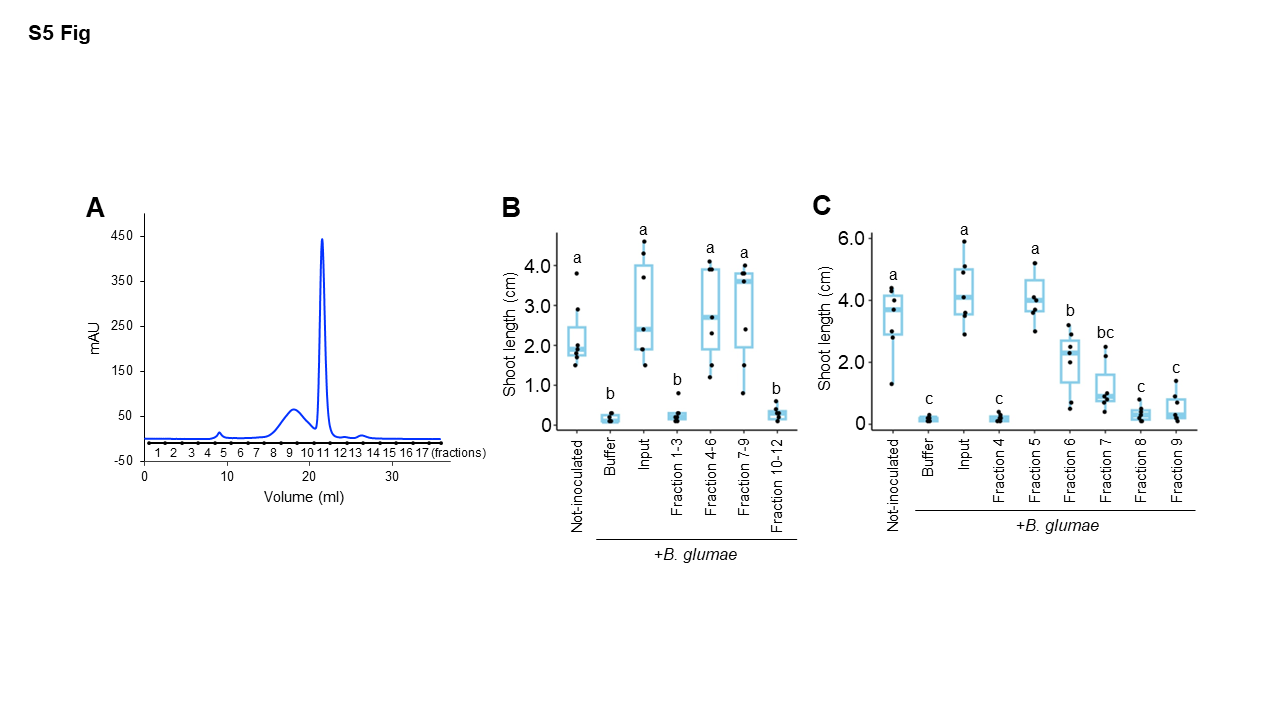

Supplement: S5 Fig — (A) Gel filtration chromatogram of the 60% saturation fractions obtained from ammonium sulfate precipitation of the culture filtrate (CF) of B. gladioli NB6-M17. (B) Rice seeds were co-inoculated with B. glumae (Bg) and the fractions obtained from the gel filtration chromatography. Three fractions were bulked for inoculation tests. The box plot shows the shoot length of rice seedlings at 7 days postinoculation (dpi): the center lines indicate the medians, the box limits represent the upper and lower quartiles, and the whiskers extend to the maximum and minimum values. Different letters indicate statistically significant differences between groups (Tukey–Kramer test, P < 0.05, n = 7). (C) Rice seeds were co-inoculated with Bg and the fractions obtained from the gel filtration chromatography. The box plot shows the shoot length of rice seedlings at 7 dpi, following the same format as in (B). Different letters indicate statistically significant differences between groups (Tukey–Kramer test, P < 0.05, n = 7). These experiments were repeated two times, and similar results were obtained. Figures show the results of one representative experiment. (TIF) [file ppat.1012645.s005.TIF]

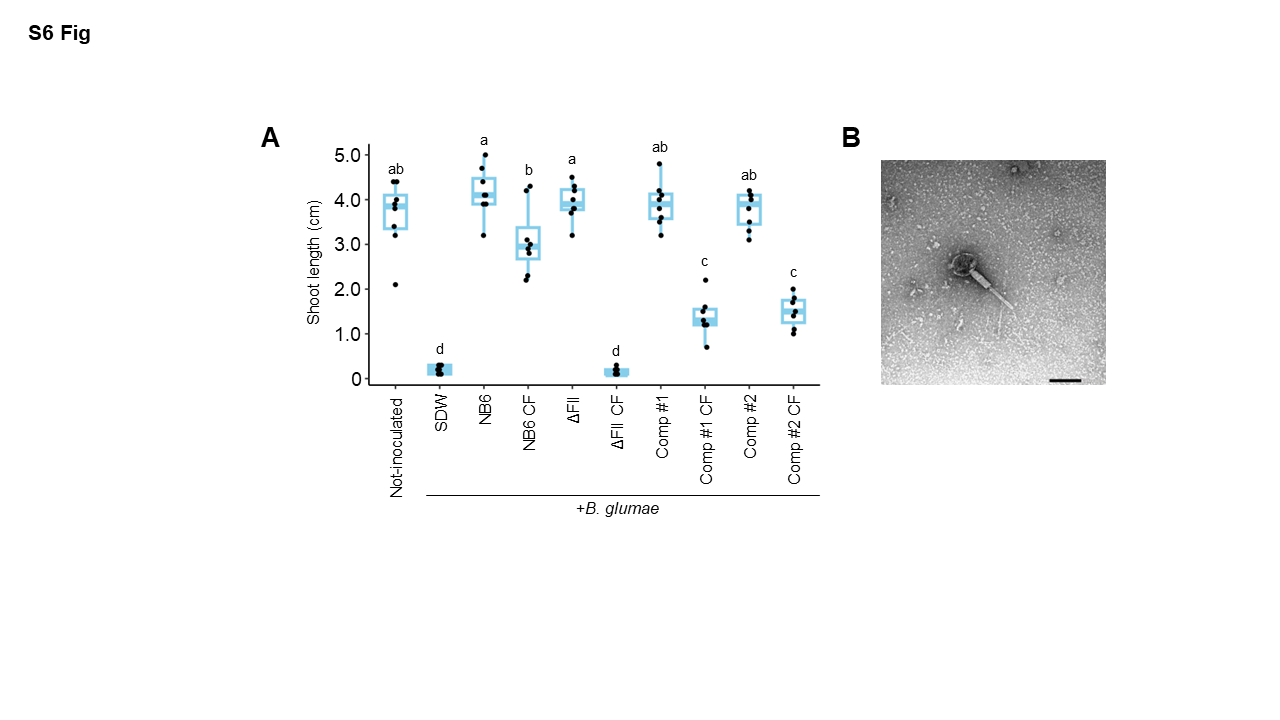

Supplement: S6 Fig — (A) FII was ectopically expressed using the pHM1 vector in ΔFII (Comp). Rice seeds were co-inoculated with B. glumae and B. gladioli NB6, ΔFII, Comp, or their respective culture filtrates. The box plot shows the shoot length of rice seedlings at 7 days postinoculation (dpi): the center lines indicate the medians, the box limits represent the upper and lower quartiles, and the whiskers extend to the maximum and minimum values. Different letters indicate statistically significant differences between groups (Tukey–Kramer test, P < 0.05, n = 8). These experiments were repeated two times, and similar results were obtained. Figure show the results of one representative experiment. (B) Picture observed in the precipitate of ΔFII via transmission electron microscopy. Scale bars indicate 100 nm. Magnification, ×50,000. (TIF) [file ppat.1012645.s006.TIF]

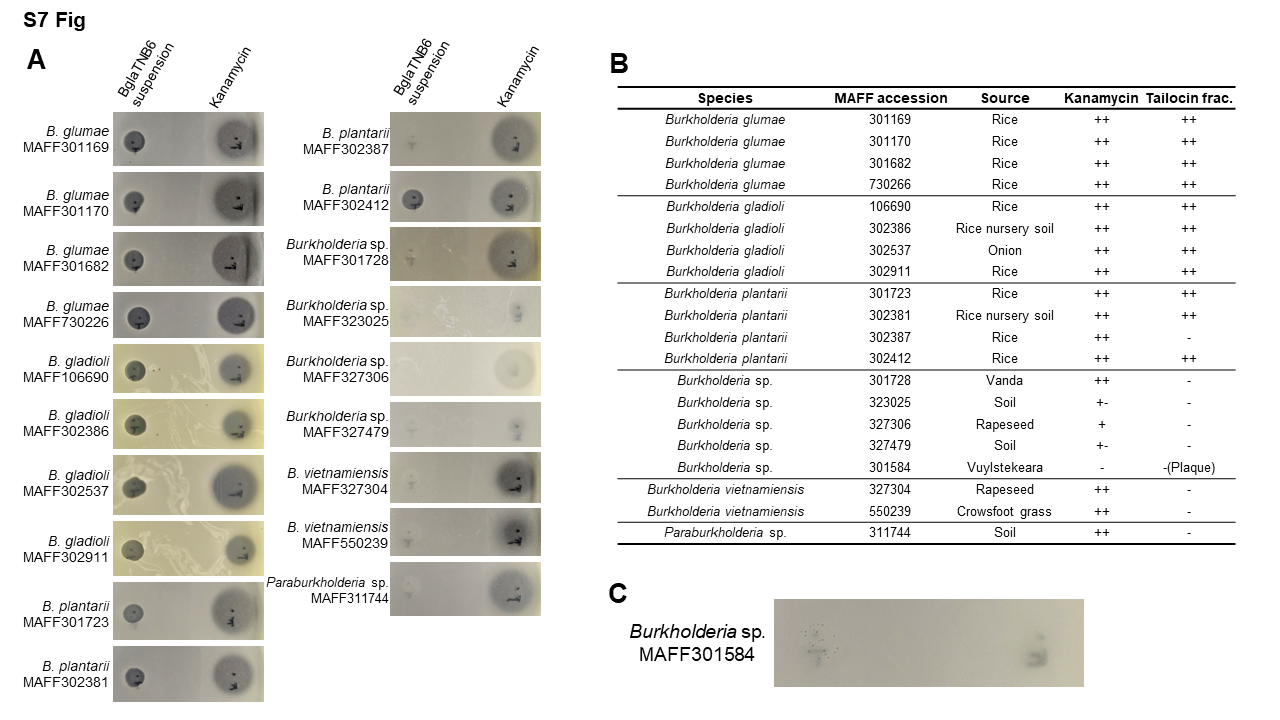

Supplement: S7 Fig — BglaTNB6 suspensions, obtained from the ultracentrifugation of the culture filtrate of B. gladioli NB6, were subjected to spot assays. Five μl of the suspensions or 0.5 mg/ml kanamycin were spotted onto LB medium containing the indicator bacterium and incubated overnight to evaluate inhibition zones. The experiment was repeated twice with similar results obtained. (A) Photos of inhibition zones formed on the LB media growing indicator bacteria. (B) Summary of the antibacterial spectrum. (C) Photo of plaques formed on the LB medium growing Burkholderia sp. MAFF301584. (TIF) [file ppat.1012645.s007.TIF]

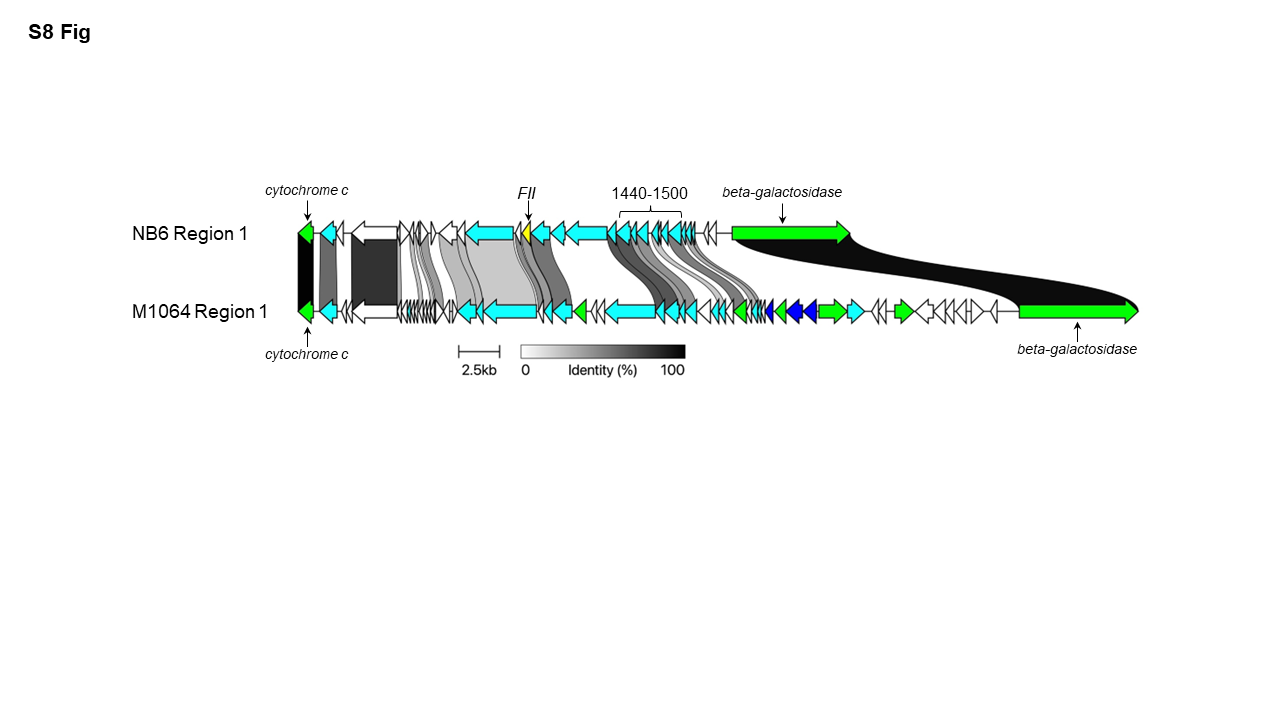

Supplement: S8 Fig — Schematic representation of the genetic organization of Region 1 prophages in the B. gladioli NB6 and M1064 genomes. Each gene is indicated by arrows, with colors based on their annotations: white for hypothetical proteins, cyan for phage-related proteins, blue for phage head proteins, and yellow for proteins identified by mass spectrometry. Chords with black-white gradation between Regions represent protein similarities. (TIF) [file ppat.1012645.s008.TIF]

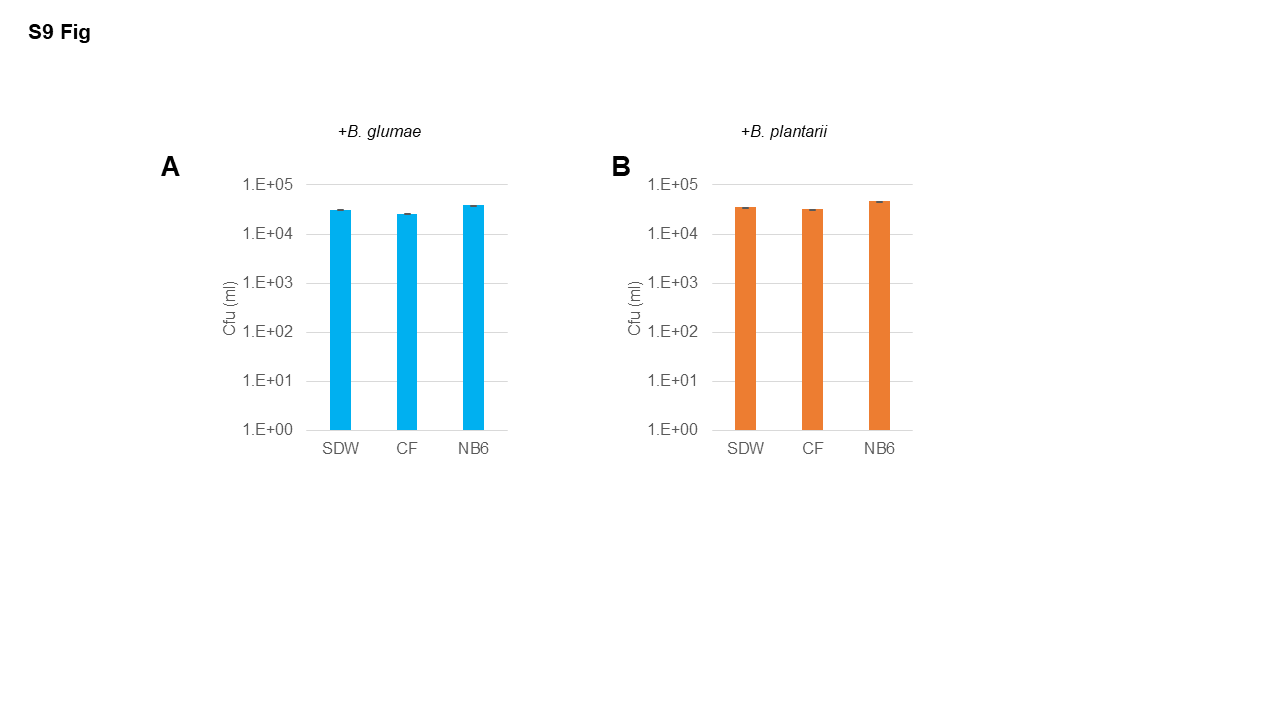

Supplement: S9 Fig — The colony-forming unit (CFU) of the excess bacterial suspension of Bg (A) and Bp (B) inocula on LB medium were measured. (TIF) [file ppat.1012645.s009.TIF]
