# Supplementary material for: BglaTNB6, a tailocin produced by a plant-associated nonpathogenic bacterium, prevents rice seed-borne bacterial diseases
Source: PLoS Pathog. 2024 Oct 18;20(10):e1012645. doi: 10.1371/journal.ppat.1012645 (PMC11524443; doi:10.1371/journal.ppat.1012645)
Supplement: Attachment — Submitted filename: Response to reviewers.pdf [file ppat.1012645.s015.pdf]

We greatly appreciate the reviewers for their invaluable comments and advice, which have significantly improved our original manuscript. Below is a list of the changes we made according to the reviewers' feedback.

Part II – Major Issues: Key Experiments Required for Acceptance

Reviewer #1: Although not the main focus of the paper they have also alluded to the host genetic control and recruitment of appropriate Burkholderia biocontrol strains as mediating Nona Bokra's tolerance to these rice pathogens. On this point the evidence is both circumstantial and unconvincing. The variation in bacterial diversity in different cultivars is interesting although there are more changes than just Burkholderia (which counters claims on Line 396) and this also ignores that these are japonica vs indica cultivars. It would be helpful to also look at the Bleiyo microbiome. Based on the authors hypothesis, Bleiyo should lack enrichment for Burkholderia.

We have added information on the bacterial strains isolated from Bleiyo as Table S1-2. In our analysis of 12 randomly selected colonies from the plate spread with the suspension of healthy, non-inoculated Bleiyo, only one Burkholderia strain was detected. These results might reinforce our speculation that the presence of Burkholderia strains in Nona Bokra supports the cultivar's tolerance. We have added this statement to the Results part, Revised L138-141: "We observed that bacterial strains belonging to the Burkholderia genus were more frequently isolated from Nona Bokra (27-35%, S1-1 Table) than from Bleiyo (8%, S1-2 Table) and Nipponbare (5-15%, S2 Fig.), and other previously examined rice cultivars such as IR-BB1, TN1 [12]". Discussion part Revised L411-413: "We observed that species belonging to the Burkholderiaceae family were more abundant in the bacterial community of Nona Bokra than in that of the susceptible cultivar Nipponbare (S2 Fig.) and Bleiyo (Table S1-2)". M&M part L497-498: "The isolated bacteria from Nona Bokra and Bleiyo were identified through 16S rRNA gene sequencing using the primers listed in Table S4".

Also, while testing in rice seedlings is interesting, the authors should conduct in vitro tests for Bg and Bp viability after being mixed with NB6, CF and tailocin using the same conditions for prepping inoculum. What is the amount of time between mixing and seed vacuum infiltration?

The rice seeds and bacterial suspension were vacuum inoculated for 5 minutes and then gently mixed for 1 hour. After removing the excess bacterial suspension from the seeds, they were sown on either MS medium or soil. These detailed inoculation methods have been added in Revised L511-512. According to the comment, we have added the results of the CFU

measurement of the excess bacterial suspension of inocula as S9 Fig, confirming their viability. We added the statement as follows "The colony-forming unit (CFU) of the excess bacterial suspension was measured and confirmed for viability (S9 Fig.)" (Revised L510-512).

I'm curious about the M17 transposon mutant. Were you actively seeking mutants with reduced protein secretion?

The M17 transposon mutant was identified through an attempt to discover the causative agent using reverse genetics. We generated numerous random mutants using transposons and screened for those with reduced plant protection effects. As a result, we obtained several mutants, one of which was the M17. This mutant showed reduced plant protection effects by the bacteria, but its CF retained the effects.

EM analysis should also be conducted of the DFII and DFII complement lines for loss and restoration of tailocin-like structures.

In the EM analysis, tailocin-like substances were not observed in the *ΔFII*. The image has been added to S6B Fig. and mentioned in the results section as "Tailocin was not observed in the case of *ΔFII* mutant (S6B Fig.)" (Revised L288-289). Since quantitative analysis by microscopic observation is difficult, we have not observed *ΔFII* complement lines that partially complemented the protective activity of the CF of *ΔFII*.

Also while BglaTNB6 makes strong contributions to restricting B. plantarii growth it is clearly not "essential".

We agree with the comment and have corrected the following sections as "greatly contributed to the biocontrol activity" (Revised L45, L335, Fig. 5, L 394).

Reviewer #2: No major issues found

Reviewer #3: -In general, I found the manuscript reasonably easy to read, however there were a number of sentences in the discussion that I found hard to interpret (I've indicated each in minor comments). In my opinion, these sentences need to be addressed.

-Although the authors acknowledge the data showing that there are at least two systems operating to suppress disease (one contact-independent tailocin and another, likely contact dependent system), this is not really addressed at all in the discussion. I think this is a more important point to discuss its implications for disease suppression and if there are other examples in plants (or elsewhere), where a biocontrol strain has multiple systems that

contribute to biocontrol.

According to the reviewer's comment, we have added the following sentence to Revised L389-394: "Some examples exist where biocontrol bacteria protect plants from pathogens through multiple mechanisms [40]. For instance, the rhizosphere bacterium Pseudomonas putida IsoF isolated from tomato possesses both the ability to directly kill pathogenic bacteria through contact-dependent inhibition (CDI) and to induce systemic resistance in the plant by producing acyl-homoserine lactone (AHL) [41,42]. "

-There should be more explicit descriptions of how genetic data is confirmed. It looks as though multiple deletion mutants were used in Figs 2, 3, and 5 as a way to confirm that the phenotypes weren't related to auxiliary mutations? However, I don't believe this is stated clearly in the text (either in methods or results section).

The method for creating the deletion strains and the primers used for PCR verification are detailed in the M&M section (Revised L565-576) as well as in the Supplementary Information (S4 Table).

Additionally, the methods section contains language indicating that a complement strain of the FII mutant was generated. I don't recall seeing this strain in the results (but I might have missed it)?

These results are shown in S6A Fig. and are described in the main text at Revised L213, indicating that partial complementation was achieved.

-L236 – This statement indicates that the capsid proteins are not present in the predicted prophage regions, however, table S3 indicates that this progaphe region was scored as intact, which I would think would be odd if the capsid genes are missing. This discrepancy should be addressed.

It is indeed puzzling that it was considered intact despite the absence of a capsid. However, this discrepancy can only be attributed to the algorithm used in this analysis method.

Part III – Minor Issues: Editorial and Data Presentation Modifications

Reviewer #1: L63: Using a mutant that secretes proteins in low abundance

We agree with the comment and have corrected the sentence as "Using a mutant that secretes proteins in low-abundance proteins" (Revised L65).

L111: why would high specificity limit emergence of tolerance in the targeted organism?

There are clear examples of mutations that allow evasion of tailocins

We agree the comment. Since tailocin-resistant pathogens can also emerge, we have removed this description (Revised L112).

L114: citation 19 "The sensitivity of phages to environmental stressors such as UV irradiation and soil (type, pH, moisture, organic matter content) is still an obstacle to phage therapy on plants" Tailocins lack nucleic acid and so should be less sensitive to UV

We agree the comment. Since reference 19 pertains to phages and tailocins are thought to be insensitive to UV light, we have removed this sentence (Revised L114-116).

L128: I see no mention of this pathogen resistance correlating with biocontrol strains in Truyens et al The Upreti and Thomas seems very limited and circumstantial.

We agree the comment. Since reference 28 discusses the presence of beneficial endophytes in seeds, we have added this point to the statement as "It has been shown that several of the seed endophytes isolated from seed possess biocontrol potential [28], and…" (Revised L128-129).

L134: Upload the 16S sequences to a public repository

The 16S sequences of the 24 bacterial strains have been deposited in DDBJ and have added the statement at the M&M section as "Those 16S rRNA gene sequences were submitted to the DNA Data Bank of Japan Sequence Read Archive (https://www.ddbj.nig.ac.jp/dra/index-e.html) under accession numbers LC831823 to LC831846" (Revised L498-501).

Fig 1D: Relative bacterial population data should be log transformed. Which strains of Bg and Bp were used in inoculation tests?

According to the comment, we have converted the graphs (Fig. 1 DE) to logarithmic scales. Additionally, to improve visibility, we have added numerical values above the bars.

L205: inhibitory effect on seedling growth

We have corrected the sentence (Revised L211). We appreciate the comment.

L227: encoded by a prophage-like region

We have corrected the sentence (Revised L235). We appreciate the comment.

L347: There's a noticeable shift in writing quality in the first paragraph of the discussion (for instance, distinct feather). Also, why is it unexpected to have a tailocin region in the genome. Where else would it be?

We have revised the first paragraph as "In the present study, we demonstrated that NB6, a nonpathogenic strain of B. gladioli (S1 Fig.), protects rice seedlings against both Bg and Bp infections (Fig. 1). Notably, NB6 almost completely restored the severe growth defects caused by these pathogens (Fig. 1). We have previously reported several promising biocontrol agents (e.g., *Pseudomonas putida* RSBs and *Pantoea dispersa* BB1) however, these agents provided significant, yet incomplete, recovery [12,15]. Therefore, we speculated that NB6 might have a distinct feature compared to the other agents" (Revised L361-367). We agree the comment, since it is expected that tailocin would be present in the genome, we have revised that part as "Because this tailocin is a phage variant that is inserted into the bacterial genome (prophage), it is implied that another microbe–microbe interaction via these phages and phage-like particles could shape the phyllosphere microbiota especially at the timing of pathogen invasions."(Revised L370-374).

L478: Which strains of Bg and Bp were used in inoculation tests?
In the inoculation tests, either *B. gladioli* or *B. plantarii* was used, so we have added "Bg and Bp" at Revised L504.

Reviewer #2: 1. To distinguish from B. plantarii, 'Bp' indicated for base pairs should be 'bp' (e.g. lines 185 and 539)
We agree the comment and have corrected the words (Revised L191, 567).

2. Line 269-315: The authors presented a TEM image of the tailocin from the CF fraction of B. gladioli NB6. To claim that the phage tail-like structure is the tailocin B6, the authors should mention or present data that no such structure was observed in the CF of the FII or region1-deleted mutant.
In the EM analysis, tailocin-like substances were not observed in the *ΔFII*. The image has been added to S6B Fig. and mentioned in the results section as "Tailocin was not observed in the case of *ΔFII* mutant (S6B Fig.)" (Revised L288-289). Since quantitative analysis by microscopic observation is difficult, we have not observed *ΔFII* complement lines that partially complemented the protective activity of the CF of *ΔFII*.

3. It is not clear why the authors mentioned 'as expected' in the sentence on line 322. How did they expect that the cell suspension of the FII-deficient mutant would also exhibit reduced protective activity unlike Bg, with the observation that B6 was effective to both Bg and Bp?
Initially, we assumed that the biocontrol effect of NB6 was solely due to tailocin. Consequently,

we expected that both the tailocin mutant culture supernatant and the live bacteria would lose their protective activity, which is why we used the phrase "as expected." However, as the reviewers commented, the differing effects on *B. plantarii* and *B. gladioli* were not anticipated. Therefore, we have removed “as expected” (Revised L334).

4. Line 334: The sentence is for Bp, not for Bg because B6 is not required for the activity of the NB6 cells. The sentence should be modified, reflecting this fact.
We agree with the comment and have added “In the case of Bp treatment,” at Revised L344.

5. Please refine the sentence on lines 350 – 353.
We have revised the first paragraph as “In the present study, we demonstrated that NB6, a nonpathogenic strain of B. gladioli (S1 Fig.), protects rice seedlings against both Bg and Bp infections (Fig. 1). Notably, NB6 almost completely restored the severe growth defects caused by these pathogens (Fig. 1). We have previously reported several promising biocontrol agents (e.g., *Pseudomonas putida* RSBs and *Pantoea dispersa* BB1) however, these agents provided significant, yet incomplete, recovery [12,15]. Therefore, we speculated that NB6 might have a distinct feature compared to the other agents” (Revised L361-367).

6. Line 396: Please provide more information of the ‘M gene’ with one or two sentences.
We have added an explanation regarding the M gene as follows, ““M gene” [45] that coded as microbiome-shaping genes in plants” at Revised L416-417.

7. Line 482: Please describe more detail about the vacuum inoculation method, such as duration of vacuum, psi, drying process before planting, etc.
The rice seeds and bacterial suspension were vacuum inoculated for 5 minutes and then gently mixed for 1 hour. After removing the excess bacterial suspension from the seeds, they were sown on either MS medium or soil. These detailed inoculation methods have been added in Revised L509-510.

8. Lines 541 and 547: Please describe how the bacterial cells were transformed. Heat shock, electroporation, or conjugation?
We have added details regarding the processing method as “by electroporation” at Revised L570.

Reviewer #3: L43-44 - This sentence should be reworded since species (not strains) are listed.
We have corrected the word (Revised L43). We appreciate the comment.

L49-50 - I don't think this sentence logically flows form the preceeding sentences, since it is not clear/established that disease suppression is connected to the competitive fitness of NB6.

As you pointed out, it is still speculative whether the suppression of disease symptoms is due to the competitive fitness between NB6 and the pathogen. Therefore, we have revised this sentence as "These results suggested that BglaTNB6 may enhance the competitiveness of NB6 against a specific range of bacteria." (Revised L49-51).

L60 – Nona Bokra was described as resistant in the abstract, but as tolerant in this line. These (and all other designations in the manuscript) should be harmonized.

We agree with the comment and have corrected the words as "resistant" (Revised L61, L132, L133).

L91-94 – This sentence seems to go in a direction not relevant to this manuscript, I think this can be removed.

Although the discovery of plant genes related to resistance in Nona Bokra is outside the main focus of this paper, we consider it an important finding and have therefore retained this description.

L135 – Was this strain only tested agains Nipponbare? Or was it tested for pathogenicity toward other rice cultivars?

We are currently confirming its effectiveness in other varieties as well and expect to report this soon.

L135 – 137 - This statement seems to require the reader to compare the number of isolates from table S1 to ref. 12, which makes it unnecessarily complicated to review this statement. This should be more supported directly in this text.

We have added the information on bacterial strains isolated from Bleiyo as Table S1-2 in accordance with the other reviewer's comment. In our analysis of 12 randomly selected colonies from the plate spread with the suspension of healthy, non-inoculated Bleiyo, only one Burkholderia strain was detected. These results might reinforce our speculation that the presence of Burkholderia strains in Nona Bokra supports the cultivar's tolerance. We have added this statement to the Results part, Revised L138-140: "We observed that bacterial strains belonging to the Burkholderia genus were more frequently isolated from Nona Bokra (27-35%, S1-1 Table) than from Bleiyo (8%, S1-2 Table) and Nipponbare (5-15%, S2 Fig.), and other previously examined rice cultivars such as IR-BB1, TN1 [12]".

L181 – 182 – How does the 60% saturation fraction confirm proteinaceous identity? Can the authors cite something to support this statement?

As shown in S3C Fig., we fractionated the samples into 20%, 40%, 60%, and 80% fractions, and tested the plant protection effects of each fraction. The results indicated that the 60% fraction exhibited the highest plant protection effect.

L226 – For this line and all similar lines related to the other figures, are the results of one representative experiment shown or are the graphs representative of all of the combined data?

All figures show the results of one representative experiment. We added the statement at Figure legend part.

L234 – Please specify what is meant by "homologies" – nt or aa identity/similarity?

In response to the inquiry about "homologies," in this context, we refer to amino acid (aa) identity/similarity with phiE202 and AP3, were classified as the most common phages of Region 1 and Region 2, respectively, based on PHAST analysis (S3 Table). We have added "amino acid (aa) identity/similarity" at Revised L242.

L241 – What genes specifically were deleted? Do these numbers refer to the locus tags? Or some other gene identifier?

These numbers refer to the locus tags of NB6.

L250 – Why compare region 1 and 2 in this manner? Do the authors think these two regions are evolutionarily related?

In the search for active fractions, proteins coded by regions 1 and 2 were identified through mass spectrometry. We then compared these regions. As the reviewer suggested, there may be a possibility of an evolutionary relationship between them.

L293-294 – This sentence seems unwarranted given the data. It might be true, but I don't think there is sufficient data to conclude this.

We agree the comment. Further examination of the spectrum of BglaTNB6 is needed, and we have added this point as "Further detailed experiments to reveal the spectrum of BglaTNB6 are necessary." (Revised L305-306).

L322 – itals deltaFII

We have corrected all the word italics. We appreciate the comment.

L336 – This figure heading should be revised, since the data shows that the BglaTNB6 contributes to biocontrol activity (but is not essential, given the other contact dependent system)

We have corrected the sentence as "BglaTNB6 greatly contributed for the biocontrol activity of *B. gladioli* NB6 against *B. plantarii*." (Revised L348). We appreciate the comment.

L352 – I don't understand this sentence

L353 – I don't understand what is meant by "distinct feather".

We have revised the first paragraph as "In the present study, we demonstrated that NB6, a nonpathogenic strain of B. gladioli (S1 Fig.), protects rice seedlings against both Bg and Bp infections (Fig. 1). Notably, NB6 almost completely restored the severe growth defects caused by these pathogens (Fig. 1). We have previously reported several promising biocontrol agents (e.g., *Pseudomonas putida* RSBs and *Pantoea dispersa* BB1) however, these agents provided significant, yet incomplete, recovery [12,15]. Therefore, we speculated that NB6 might have a distinct feature compared to the other agents" (Revised L361-367).

L357-358 – This sentence doesn't make sense to me since tailocins are by definition phage genomes that have inserted into the bacterial genome. What makes this "unexpectedly"?

L358-359 – I don't get the logic of this clause.

We agree the comment, since it is expected that tailocin would be present in the genome, we have revised that part as "Because this tailocin is a phage variant that is inserted into the bacterial genome (prophage), it is implied that another microbe–microbe interaction via these phages and phage-like particles could shape the phyllosphere microbiota especially at the timing of pathogen invasions."(Revised L370-374).

L361-363 – What is "it" in this sentence?

We are referring to the recombinant rFII protein. To clarify this point, we have made the revisions as "While recombinant FII protein (rFII) itself did not show protective activity," at Revised L377.

L383 – What tailocin loci don't originate from prophages that would necessitate "some" for this sentence.

We agree with the comment and have removed "some" at Revised L403.

L386-388 – This sentence seems out of place for what is being reported, I suggest removing.

Since we choose the Bleiyo cultivar because it is resistant cultivar according to the previous

report (reference 30), we retain the sentence.

L414 – How does this figure confirm this statement?

As you pointed out, since we did not perform a comparison with the cited references in this figure, we have removed that part and added "(S7 Fig.)" after BglaTNB6 (Revised L439).

L435 – It should be indicated that Rhizoviticin is a tailocin.

The fact that Rhizoviticin is a tailocin is stated at Revised L443. For clarity, we have added "(F-type tailocin) "at this section (Revised L458).
