# Supplementary material for: BglaTNB6, a tailocin produced by a plant-associated nonpathogenic bacterium, prevents rice seed-borne bacterial diseases
Source: PLoS Pathog. 2024 Oct 18;20(10):e1012645. doi: 10.1371/journal.ppat.1012645 (PMC11524443; doi:10.1371/journal.ppat.1012645)
Supplement: Attachment — Submitted filename: Response to re-reviewers.docx [file ppat.1012645.s016.docx]

We greatly appreciate the reviewers for their invaluable comments and advice, which have significantly improved our original manuscript. Below are the changes we made according to the reviewer’s feedback.

**Part III – Minor Issues: Editorial and Data Presentation Modifications**

**Please use this section for editorial suggestions as well as relatively minor modifications of existing data that would enhance clarity.**

**Reviewer #3: It would still be good for the authors to state explicitly in the text (either figure legends, results section, or methods section) that the multiple mutant isolates shown in figures 2, 3, and 5 are all included to confirm that the phenotype of the mutants is related to the deletion that was introduced, and not a spurious mutation that was picked up during the mutant generation process. I think this is why multiple mutant isolates are included in the figures, but it isn't actually confirmed in any text.**

In response to the reviewer’s comment, we have added the following statement: “By using two independent mutant strains in the experiments, we confirmed that the observed phenotype was not due to a spurious mutation arising during the mutant generation process” (Revised lines 568-570). Additionally, to clarify the use of two independent mutants, we have modified the figure legends as follows:

Fig 2. “(D and E) Rice seeds were co-inoculated with Bg and NB6, along with its two independent gene-disruptive mutants (*ΔFII* #3 and #8, *Δcap* #4 and #11, and *ΔporA* #6 and #8), their respective culture filtrates (CFs), or sterilized distilled water (SDW).”

Fig 3. “(B) Rice seeds were co-inoculated with *B. glumae* (Bg) and NB6, along with its two independent gene-disruptive mutants (*Δ1440-1500* #4 and #5), their respective culture filtrates (CFs), or sterilized distilled water (SDW).”

Fig 5. “Rice seeds were co-inoculated with *B. plantarii* (Bp) and *B. gladioli* NB6, along with its two independent gene-disruptive mutants (*ΔFII* #3 and #8), their respective culture filtrates (CFs), or sterilized distilled water (SDW).”
